# Discordant and false-negative interpretations at digital breast tomosynthesis in the prospective Oslo Tomosynthesis Screening Trial (OTST) using independent double reading

**DOI:** 10.1007/s00330-023-10400-0

**Published:** 2023-11-08

**Authors:** Per Skaane, Bjørn Helge Østerås, Stanimir Yanakiev, Terese Lie, Ellen B. Eben, Randi Gullien, Siri H. B. Brandal

**Affiliations:** 1 Division of Radiology and Nuclear Medicine, Department of Breast Diagnostics, Oslo University Hospital, University of Oslo, Oslo, Norway; 2 Institute of Clinical Medicine, University of Oslo, Oslo, Norway; 3 Department of Physics and Computational Radiology, Oslo University Hospital, Oslo, Norway

**Keywords:** Breast cancer screening, Double reading, Interobserver variability, Digital breast tomosynthesis, Mammography screening

## Abstract

**Objectives:**

To analyze discordant and false-negatives of double reading digital breast tomosynthesis (DBT) versus digital mammography (DM) including reading times in the Oslo Tomosynthesis Screening Trial (OTST), and reclassify these in a retrospective reader study as missed, minimal sign, or true-negatives.

**Methods:**

The prospective OTST comparing double reading DBT vs. DM had paired design with four parallel arms: DM, DM + computer aided detection, DBT + DM, and DBT + synthetic mammography. Eight radiologists interpreted images in batches using a 5-point scale. Reading time was automatically recorded. A retrospective reader study including four radiologists classified screen-detected cancers with at least one false-negative score and screening examinations of interval cancers as negative, non-specific minimal sign, significant minimal sign, and missed; the two latter groups are defined “actionable.” Statistics included chi-square, Fisher’s exact, McNemar’s, and Mann–Whitney *U* tests.

**Results:**

Discordant rate (cancer missed by one reader) for screen-detected cancers was overall comparable (DBT (31% [71/227]) and DM (30% [52/175]), *p *= .81), significantly lower at DBT for spiculated cancers (DBT, 19% [20/106] vs. DM, 36% [38/106], *p *= .003), but high (28/49 = 57%, *p *= 0.001) for DBT-only detected spiculated cancers. Reading time and sensitivity varied among readers. False-negative DBT-only detected spiculated cancers had shorter reading time than true-negatives in 46% (13/28). Retrospective evaluation classified the following DBT exams “actionable”: three missed by both readers, 95% (39/41) of discordant cancers detected by both modes, all 30 discordant DBT-only cancers, 25% (13/51) of interval cancers.

**Conclusions:**

Discordant rate was overall comparable for DBT and DM, significantly lower at DBT for spiculated cancers, but high for DBT-only detected spiculated lesions. Most false-negative screen-detected DBT were classified as “actionable.”

**Clinical relevance statement:**

Retrospective evaluation of false-negative interpretations from the Oslo Tomosynthesis Screening Trial shows that most discordant and several interval cancers could have been detected at screening. This underlines the potential for modern AI-based reading aids and triage, as high-volume screening is a demanding task.

**Key Points:**

*• Digital breast tomosynthesis (DBT) screening is more sensitive and has higher specificity compared to digital mammography screening, but high-volume DBT screening is a demanding task which can result in high discordance rate among readers.*

*• Independent double reading DBT screening had overall comparable discordance rate as digital mammography, lower for spiculated masses seen on both modalities, and higher for small spiculated cancer seen only on DBT.*

*• Almost all discordant digital breast tomosynthesis-detected cancers (72 of 74) and 25% (13 of 51) of the interval cancers in the Oslo Tomosynthesis Screening Trial were retrospectively classified as actionable and could have been detected by the readers.*

## Introduction

Digital breast tomosynthesis (DBT, 3D) has emerged as a new screening technique since it has potential to resolve limitations of conventional digital mammography (DM, 2D). DBT showed reduced recall rates especially in retrospective US studies and increased cancer detection in prospective European trials using double reading [1]. Meta-analysis has shown little evidence of a difference between DBT and DM in interval cancer rate [2], but a recent trial reported reduced rate in DBT screening [3].

Missed cancers are caused by detection (perception) or interpretation (classification) error [4], both representing a challenge in high-volume screening using batch reading. Studies have shown improved interreader reliability using DBT compared to DM, with increased confidence for architectural distortion, a commonly missed abnormality at screening [5]. DM studies reported about 50% of interval cancers are visible on prior screening mammograms, of which 30% are “minimal sign” lesions and 20% false-negatives [6]. Population-based 2D screening studies found 23% of cancers detected by only one of two readers [7]. One prospective DBT study reported a significant decrease in discordant recalls for cancers, suggesting that usefulness of double reading is reduced using DBT [8]. However, the challenges of perception and interpretation errors are potentially greater in DBT than in DM screening due to more images, complex hanging protocols, and long interpretation times with reader fatigue.

There is a lack of knowledge from double reading DBT versus DM screening regarding discordant (cancer missed by one reader) and false-negative (cancer missed by both readers) interpretations. The aim of our study was to compare discordant and false-negatives, including reading times, in high-volume screening using batch reading.

## Materials and methods

The Oslo Tomosynthesis Screening Trial (OTST) was approved by the regional ethical committee (*clinicaltrials.gov*, NCT01248546). Hologic sponsored the study by providing equipment and financial support for additional image evaluation. The authors had full control of all data. OTST results have been reported [9–14]. This article presents unpublished data.

### Study participants

The prospective OTST invited women age 50–69 to two-view mammography. During the study period (November 22, 2010, to December 19, 2012), 59,009 women were invited and 34,740 (58.9%) attended. Women were asked to participate and undergo DBT in addition to DM, if there was availability of radiographers and imaging systems. Women with pacemakers, disabled women unable to stand, and women with implants were excluded. A total of 24,301 women were included in the OTST.

### Imaging procedures

Examinations were performed using Hologic Dimensions systems using standard screening exposure control (“auto filter”). Craniocaudal and mediolateral oblique views of each breast were obtained using combo mode (same compression DM and DBT).

### Training and image evaluation

Eight radiologists (P.S., E.E.) participated in the OTST, and received training in DBT interpretation using fixed trial hanging protocols 2 weeks prior to the OTST. Training included 100 screening exams enriched with cancers. The radiologists had 2–31 years’ experience in screening mammography.

Each screening exam was independently interpreted in parallel by four different radiologists in batch mode (usually 60 to 80 women per day) using four dedicated workstations, one for each trial arm: (1) DM; (2) DM plus computer-aided detection (CAD) (ImageChecker 9.3, Hologic); (3) DBT plus DM; and (4) DBT plus synthetic mammography (SM). Balanced assignment of radiologist with respect to arm was difficult in daily practice. After closing their session, radiologists had no access to other readers’ ratings. Each radiologist rated exams per breast using the 5-point scale for probability of cancer implemented in the Norwegian program: 1 = negative or definitely benign; 2 = probably benign; 3 = indeterminate; 4 = probably malignant; and score 5 = malignant. Scores 2–5 are positive. We defined “discordant miss” as screening-detected cancer with highly suspicious score 4 or 5 by one radiologist and missed (score 1) by second reader. Double reading was considered positive if either of the constituent arms (DM: arm 1 or 2, DBT: arm 3 or 4) were positive.

Mammographic findings (circumscribed mass, spiculated mass, architectural distortion, calcifications ± density) were specified for each positive score. Spiculated mass and architectural distortion are merged in our analyses as spiculated cancers. Scores were recorded directly into the national screening database and locked after each session. Interpretation time was recorded automatically.

Examinations with at least one score of 2 or greater were discussed at a consensus-based meeting (minimum two readers participating) with all data available with a decision to dismiss or invite for diagnostic work-up. Consensus meeting was free to make their decision, but in general all exams with score 4 or 5 were recalled. Short-term follow-up was not given. Breast density (BI-RADS 4th edition) was given at consensus meeting (5th edition not used at beginning of the OTST). Default hanging protocols were preset for all arms, including 4 steps for both DM arms and 8 steps for both DBT arms. All readers used manual scrolling and rarely included slabs.

### Histopathology and definitions

Cancers (*n *= 230) were confirmed through pathology. Follow-up was 24 months from screening. Cases were verified as negative by querying the national cancer registry. Interval cancer was defined as malignancy after negative screening before next scheduled examination.

### Reclassification of screening examinations

Screening examinations (DM and DBT) of all screening-detected cancers with at least one false-negative score (*n *= 130) and all interval cancers (*n *= 51) were mixed with normal/benign cases (*n *= 59). In a retrospective reader study carried out more than 6 years after the OTST (in March 2019), four radiologists (S.Y., T.L., E.E. (participated in the OTST), S.B.) independently reviewed these exams (blinded to trial interpretations). If a suspicious lesion was found, the reader had to specify localization and mammographic findings and give a malignancy score using the same 5-point rating scale. Readers first analyzed DM and gave a conclusion before DBT examination was reviewed. A final consensus session, including all four readers and with all information available, finally classified findings as follows: negative (cancer in retrospect not visible); non-specific minimal sign (cancer visible but subtle features); significant minimal sign (findings suspicious of cancer); and false-negative (“missed cancer”). The two latter groups were defined as “actionable,” i.e., cancer should have been detected at screening.

### Statistical analyses

Comparison of unpaired ratios was performed (B.H.Ø.) using the chi-squared test or using Fisher’s exact test if expected counts were low. If the ratios were paired, comparison was carried out using McNemar’s test. Comparison of reading times was performed using the Mann–Whitney *U* test. A *p*-value *p* ≤ .05 was considered statistically significant. The analysis was performed using Stata 17 (StataCorp).

## Results

### Women included

**Fig. 1:**
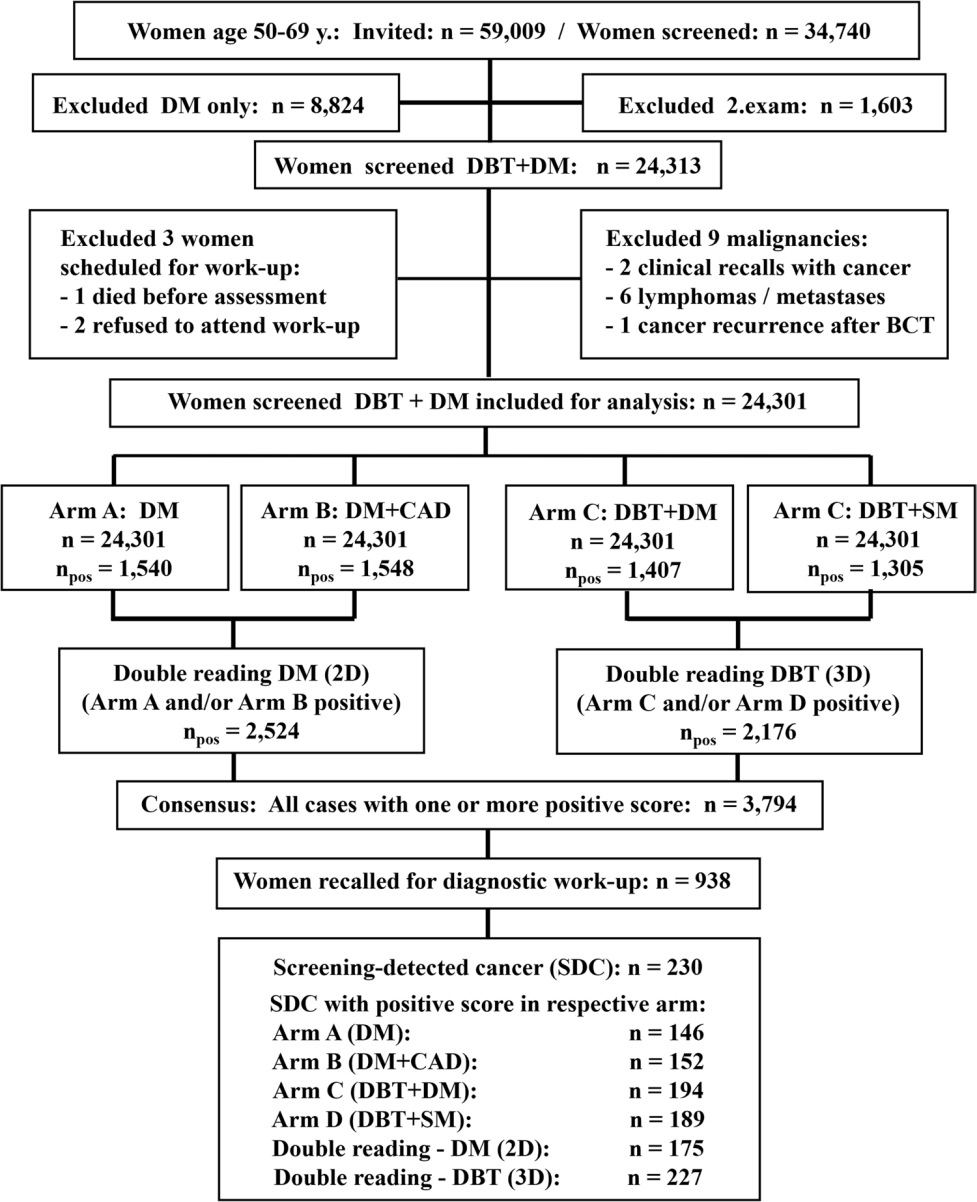
Flowchart shows number of women excluded and included for analysis, and the number of screening-detected cancer at double reading digital mammography (DM) and double reading digital breast tomosynthesis (DBT) in the Oslo Tomosynthesis Screening Trial (OTST). CAD, computer-aided detection; SM, synthetic mammography; *n*_pos_, number of positive scores

Among 34,740 attending women, the following were excluded: 8824 women underwent DM only, six with non-cancer malignancies, two with palpable cancer, one with local recurrence, all second exams of 1603 women attending twice, and three women scheduled for recall that did not return for work-up. Hence, 24,301 women represent the study population (Fig. 1).

### Screening-detected cancers

**Fig. 2:**
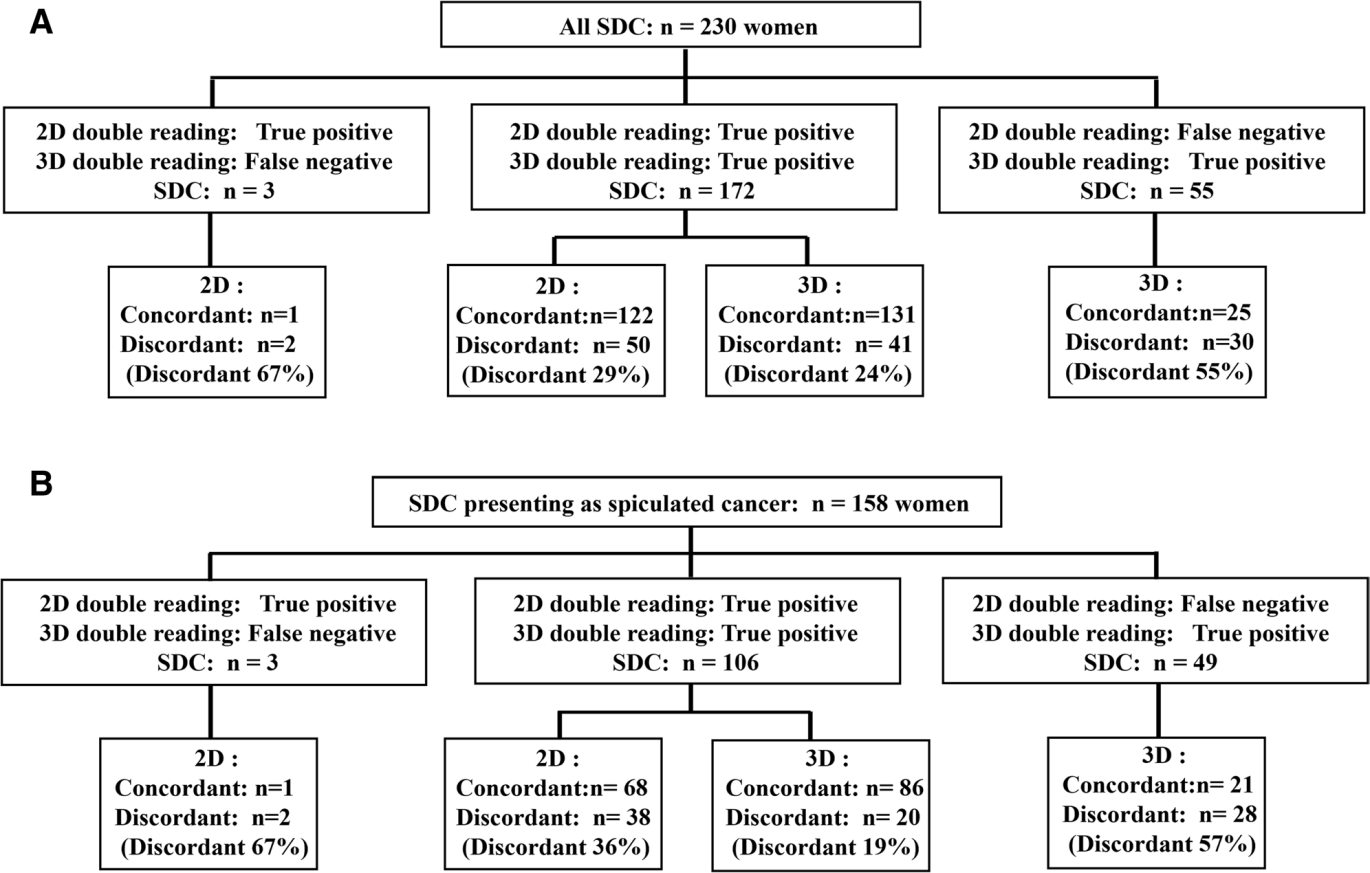
**A**, **B** Flowchart comparing results of double reading digital mammography (DM or 2D) versus double reading digital breast tomosynthesis (DBT or 3D). **A** For all 230 screening-detected cancers (SDC). **B** For 158 SDC presenting as spiculated cancer (spiculated mass or architectural distortion). True-positive: both readers (concordant) or one reader (discordant) had true-positive score. False-negative: both readers had negative score

A total of 20,507 (84.4%) of 24,301 women had a negative score in all four arms and 3794 (15.6%) a positive score in at least one arm, of which 2856 (75.3%) were dismissed and 938 recalled (all arms recall 3.9%) at consensus. Screening-detected cancers were diagnosed in 230 women of which four had bilateral cancer (Fig. 1). DBT double reading detected 227 cancers (arm C 194, arm D 189, cancer detection rate 9.3 per 1000 exams) and DM double reading detected 175 cancers (arm A 146, arm B 152, cancer detection rate 7.2 per 1000 exams) (relative increase 18.5%; McNemar, *p* < 0.001). Three cancers were detected on DM only, 172 on DBT and DM, and 55 on DBT only (Fig. 2A).

### Discordant and false-negative interpretations

A total of 130 of 230 women with screening-detected cancers had a false-negative score in at least one arm (Table 1). Overall discordant rate was 31% (71/227) for DBT and 30% (52/175) for DM (chi-squared, *p* = .81). The difference was non-significant (McNemar, *p *= .27) at DBT (24% [41/172]) versus DM (29% [50/172]) for cancers detected at both reading modes (Fig. 2A). Number of false-negatives and consequently relative sensitivity varied among readers and modality (Table 2).

**Table 1:** Histology and mammographic findings of concordant and discordant screening-detected cancers in the Oslo Tomosynthesis Screening Trial (OTST)

	All (*n *= 230)	Conc. (*n *= 100)	FN score in at least one arm (*n* = 130)							
			Disc.		DM			DBT		
			*n*	%	−/−	+/−	+/+	−/−	+/−	+/+
All	230	100	130	57	55*	52	23	3	71	56
Histology										
DCIS	36	16	20	56	3	9	8	0	14	6
IDC	83	42	41	49	16	18	7	0	26	15
IDC + DCIS	58	28	30	52	15	11	4	0	14	16
ILC	30	8	22	73	11	9	2	2	12	8
Tubular carcinoma	19	5	14	74	9	4	1	1	3	10
Other carcinoma	4	1	3	75	1	1	1	0	2	1
Mammographic features										
Circumscribed mass	11	8	3	27	0	0	3	0	3	0
Spiculated mass	138	51	87	63	43	35	9	2	44	41
Architectural distortion	20	9	11	55	6	5	0	1	4	6
Calcification with or without density	61	32	29	48	6	12	11	0	20	9

Concordant (Conc.) screening-detected cancers: true-positive (TP) score in both arms at double reading 2D (digital mammography DM) or at double reading 3D (digital breast tomosynthesis DBT) indicated with “+/+.” Discordant (Disc.) screening-detected cancers: one TP and one false-negative (FN) score at double reading indicated with “+/−.” Overlooked (missed) cancers are those with FN in both arms (“−/−”). *DCIS*, ductal carcinoma in situ; *IDC*, invasive ductal carcinoma; *ILC*, invasive lobular carcinoma

*Nine DBT-only detected cancers (manifesting on DBT as spiculated mass *n *= 6 or architectural distortion *n *= 3) were invisible on DM

**Table 2:** Screening examinations and screening-detected cancers (SDC) interpreted by each reader and each arm in the Oslo Tomosynthesis Screening Trial (OTST)

Radiologist	DM arms (2D) (arm A + B)					DBT arms (3D) (arm C + D)				
	Number of exams			Rel. sens.		Number of exams			Rel. sens.	
	DM (arm A)	DM + CAD (arm B)	SDC	TP/SDC		DBT + DM (arm C)	DBT + SM (arm D)	SDC	TP/SDC	
	*n*	*n*	*n*	*n*	%	*n*	*n*	*n*	*n*	%
Reader 1	4643	4675	86	53/86	62	5262	5318	105	92/105	88
Reader 2	981	663	20	19/20	95	663	564	17	15/17	88
Reader 3	5062	5220	89	48/89	54	3582	3530	63	46/63	73
Reader 4	2959	3103	59	39/59	66	3247	3412	62	50/62	81
Reader 5	3706	3797	67	45/67	67	4258	4169	87	78/87	90
Reader 6	1905	1895	35	22/35	63	1942	2164	34	20/34	59
Reader 7	2062	1985	35	28/35	80	2656	2456	48	41/48	85
Reader 8	2983	2907	68	44/68	65	2691	2688	44	41/44	93
All readers	24,301	24,245*	459	298/459*	65	24,301	24,301	460	383/460	83

Relative sensitivity (Rel. sens., percentage of true-positive scores TP among interpreted SDC) is presented for double reading DM (2D) and DBT (3D)

*One reader (excluded) interpreted only one batch (*n *= 56) in the DM + CAD arm including one SDC, hence 459 SDC in DM (2D). *DM*, digital mammography; *CAD*, computer-aided detection; *DBT*, digital breast tomosynthesis; *SM*, synthetic mammography

### Mammographic features and histology

Stratification by mammographic features (Table 1) showed that 158 screening-detected cancers presented as spiculated lesion, and DBT detected 98% (155/158) versus DM 69% (109/158) (McNemar, *p*<.001). Discordant rate overall for spiculated cancers was 31.0% (48/155) for DBT versus 36.7% (40/109) for DM (chi-squared, *p* = 0.33), but for 106 spiculated cancers detected at both modes (Fig. 2B), discordant rate for DBT 19% (20/106) was significantly lower than DM 36% (38/106) (McNemar, *p* = 0.003). Discordant rate for DBT-only detected spiculated cancers was significantly higher (57%: 28/49, chi-squared *p * = 0.001) compared to DBT for those detected using both modes (Fig. 2B). “Discordant miss” was seen more often in DBT-detected cancers (9% [20/227]) than in DM-detected (3% [6/175]) cancers (Fisher’s exact, *p * = 0.04). For DBT-only detected spiculated cancers (*n *= 49), the “discordant miss” rate was 16.3% (8 of 49 tumors).

For 49 DBT-only detected spiculated cancers, mean breast density was comparable for the concordant group (*n *= 21; mean score, 2.62) and the discordant group (*n *= 28; mean score, 2.57). Impact of breast density on performance in the OTST has been published [13].

**Fig. 3:**
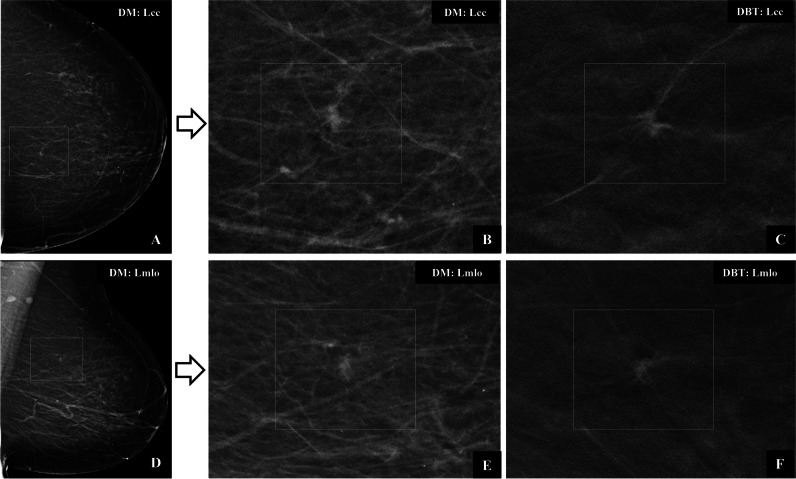
Screening images of a 67-year-old woman with a cancer in the left breast detected by digital breast tomosynthesis (DBT) only in arm C (DBT + digital mammography). **A** Craniocaudal (CC) and (**D**) mediolateral oblique (MLO) images show a small nonspecific density posteriorly (box) among several others in the breast. Zoomed DM images (**B**, **E**) show a nonconclusive small mass but zoomed DBT images (**C**, **F**) demonstrate a small spiculated mass highly suggestive of cancer. Histology: invasive ductal carcinoma 7 mm, grade 3, no axillary lymph node metastases

Most missed screening-detected cancers were invasive ductal carcinomas with or without ductal carcinoma in situ (Table 1). DBT detected significantly more invasive lobular cancers than DM (28/30 vs. 19/30, McNemar’s, *p* = 0.02). Three cancers missed at DBT but detected at DM included two invasive lobular cancers and one tubular carcinoma. Spiculated lesions and cancers with calcifications were dominant among discordant cancers (Fig. 3). No cancer presenting with calcifications was missed at double reading DBT but discordant rate was 33% (20/61).

### Reading time

**Fig. 4:**
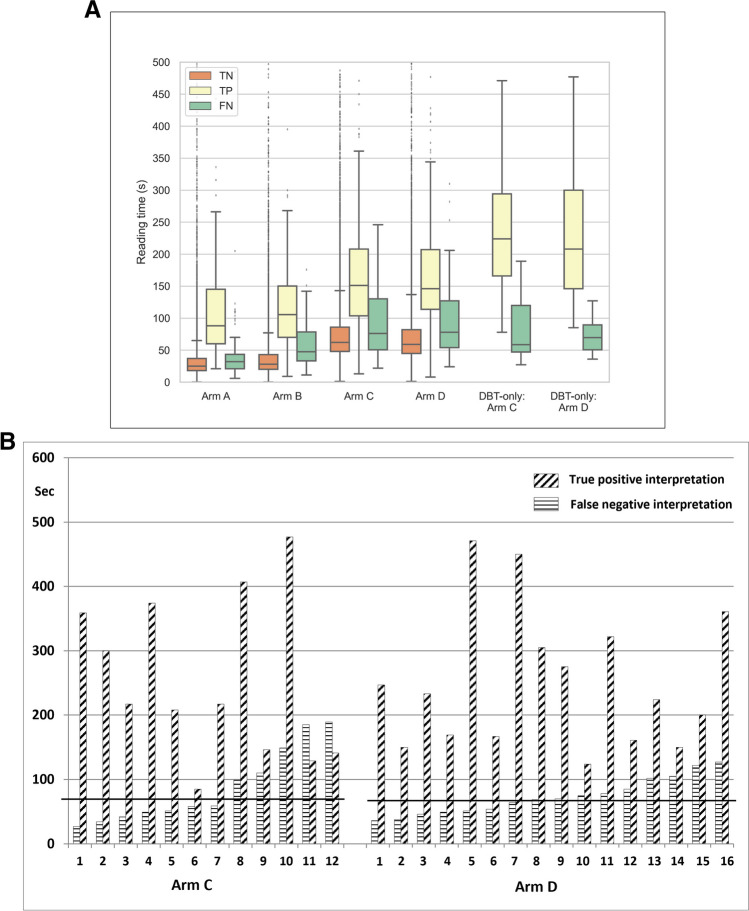
**A** Boxplot showing reading time with interquartile range for true-negative (TN), true-positive (TP), and false-negative (FN) interpretations in the four arms (arm A: digital mammography DM; arm B: DM + CAD; arm C: digital breast tomosynthesis DBT + DM; arm D: DBT + synthetic mammography SM), and for TP and FN interpretations of DBT-only detected spiculated cancers. Median reading time for TN interpretation in all four arms (*n *= 20,140): arm A = 25 s; arm B = 28 s; arm C = 62 s; arm D = 58 s. The number of TP/FN in the four arms: arm A 146/84; arm B 152/78; arm C 194/36; and arm D 189/41, respectively. Numbers of TP/FN for DBT-only detected spiculated cancers (*n *= 49) are as follows: arm C 37/12 and arm D 33/16. Outliers are indicated by points above the quartile range. **B** Bar graph showing reading time (seconds) for the 28 discordant pairs of DBT-only detected spiculated masses and architectural distortions, including 12 false-negatives in arm C and 16 in arm D. Seven (7/12 or 58%) of FN in arm C and six (6/16 or 38%) of FN in arm D had shorter reading times than the median reading time for TN interpretation (indicated by horizontal lines)

Median reading time for true-negatives (2 years follow-up) given score “1” by all readers was 25 and 28 s for DM and DM + CAD, and 62 and 58 s for DBT plus DM and DBT + SM, respectively. There was great variation for true-negatives and false-negatives among readers (Table 3). Reading times for true-positives were longer for both DM and DBT, 88 and 106 s for DM and DM + CAD, and 151 and 146 s for arm DBT plus DM and DBT + SM, respectively (Fig. 4A). False-negative reading times were longer than true-negatives, 48 versus 32 s for DM (arms A and B) and 78 versus 76 s for DBT (arms C and D) (Fig. 4A). Proportions of false-negative scores with shorter interpretation time than for true-negatives were comparable for both reading modes, 20% (10/50) for DM (arms A and B) and 27% (11/41) for DBT (arms C and D) (chi-squared, *p* = 0.54). For DBT-only detected spiculated cancers, the difference between median reading time for true-positives and false-negatives was 224 vs. 58.5 s (*p*<.001, Mann–Whitney *U* test) for arm C and 208 vs. 69.5 s (*p *< 0.001) for arm D (Fig. 4A). Individual pairs of discordant DBT-only detected spiculated cancers revealed large differences of reading times, with 46% (13/28) of tumors classified “actionable” observed as having false-negative times shorter than the median reading time for true-negatives (Fig. 4B).

**Table 3:** Median interpretation time (seconds) for true-negative and for false-negative interpretations by each reader and for each arm

Reader	Digital mammography (2D) Median interpretation time (s)						Digital breast tomosynthesis (3D) Median interpretation time (s)					
	TN reading		False-negative reading				TN reading		False-negative reading			
	DM	DM + CAD	DM		DM + CAD		DBT + DM	DBT + SM	DBT + DM		DBT + SM	
	Sec	Sec	*N* (FN/all)	Sec	*N* (FN/all)	Sec	Sec	Sec	*N* (FN/all)	Sec	*N* (FN/all)	Sec
Reader 1	20	23	16/41	29	17/45	44	57	55	9/68	68	4/37	72
Reader 2	48	81	0/8	-	1/12	142	170	156	1/8	194	1/9	127
Reader 3	30	33	21/48	41	20/41	49	86	89	5/30	131	12/33	129
Reader 4	33	38	7/19	36	13/40	79	77	68	5/27	123	7/35	94
Reader 5	25	27	14/40	30	8/27	41	55	53	3/36	42	6/51	52
Reader 6	17	20	6/16	25	7/19	15	36	36	8/17	46	6/17	47
Reader 7	37	45	4/18	73	3/17	103	71	69	3/23	125	4/25	80
Reader 8	14	17	16/40	27	8/28	44	46	36	2/21	47	1/23	253
All	25	28	84/230	32	77/229*	48	62	58	36/230	76	41/230	78

TN = true-negative score in all four arms (*n *= 20,140). *DM*, digital mammography; *CAD*, computer-aided detection; *DBT*, digital breast tomosynthesis; *SM*, synthetic mammography

*One radiologist (excluded from analysis) read only one session (arm B) which included one cancer, thus 77/229 cancers in arm B

### Retrospective classification of discordant and interval cancers

Among screening-detected cancers detected in both modes (*n* = 172), 44 of 50 (88%) discordant DM and 39 of 41 discordant DBT (95%) were considered “actionable” (chi squared, *p* = 0.80; Table 4). All three screening-detected cancers missed at DBT and all 30 discordant DBT-only detected cancers were classified as “actionable” (Table 4).

**Table 4:** Retrospective classification of discordant and false-negative cancers

Retrospective classification	DM: TP / DBT: FN *n* = 3		DM: TP / DBT: TP *n* = 172		DM: FN / DBT: TP *n* = 55		Interval cancers *n* = 51	
	DM Disc. = 2	DBT FN = 3	DM Disc.= 50	DBT Disc.= 41	DM FN = 55	DBT Disc. = 30	DM FN = 51	DBT FN = 51
Negative (cancer not visible)	0	0	0	0	9	0	35	29
Non-specific minimal sign	2	0	6	2	19	0	10	9
Significant minimal sign	0	3	36	9	26	4	5	9
Overlooked (“missed”)	0	0	8	30	1	26	1	4
All	2	3	50	41	55	30	51	51
“Actionable” (*n*)	0	3	44	39	27	30	6	13
“Actionable” (%)	0%	100%	88%	95%	49%	100%	12%	25%

Discordant (Disc)=missed by one and false-negative (FN) missed by both readers at double reading screening examinations among the 230 screening-detected cancers and the 51 interval cancers. *DM* (2D), double reading digital mammography; *DBT* (digital breast tomosynthesis), double reading DBT (3D). Retrospective classification included four groups, and the two latter (significant minimal sign and overlooked) are merged as “actionable” (i.e., these cancers should have been detected at screening)

TP = cancer detected at independent double reading, i.e., by one or both readers

Cancer was not visible on both 2D views in 22% (35/158) of screening-detected spiculated cancers. Discordant DBT interpretation occurred in 50% (6/12) when cancer was not seen on 2D mediolateral oblique view, in 64% (9/14) when not seen on 2D craniocaudal view, and in 78% (7/9) when not seen on neither craniocaudal nor mediolateral oblique view. All 35 DBT exams were identified as “actionable” (8 significant minimal sign and 27 “missed cancer”). DBT was concordant true-positive in 21 and discordant in 28 of 49 DBT-only detected spiculated cancers. DM was retrospectively grouped normal/non-specific minimal sign in 43% (9/21) of concordant and in 61% (17/28) of discordant DBT cases.

**Fig. 5:**
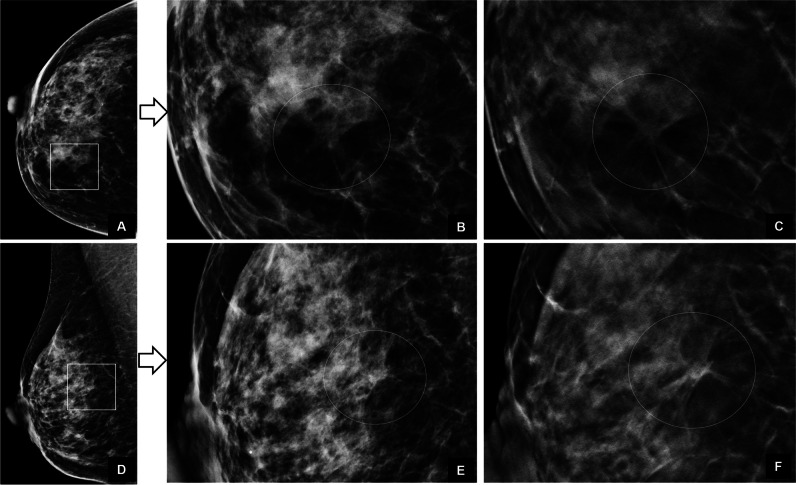
Screening images of a 67-year-old woman with interval cancer 6 months later. Digital mammography (DM) and digital breast tomosynthesis (DBT) screening examinations of the right breast are presented. Readers in all four arms gave a normal score. **A**–**C** Craniocaudal (CC) and (**D**–**F**) mediolateral oblique (MLO) views. Reader in all four arms gave a normal score. Zoomed DM images show a suspicious finding on CC view (**B**) but normal findings on MLO view (**E**) (circle). Zoomed DBT images (**C**, **F**) demonstrate a spiculated mass consistent with cancer on both views (circle). Screening examination was retrospectively classified as non-specific minimal sign at DM and as missed cancer at DBT. Histology: multifocal invasive ductal carcinoma (foci 11, 5, and 5 mm), grade 2, and two axillary lymph nodes metastases

Interval cancer was diagnosed in 51 women, and scores were negative (“1”) in all four arms in 36 of these (Fig. 5). Retrospective classification (Table 4) identified 12% (6/51) using DM and 25% (13/51) using DBT as “actionable” (McNemar, *p* = 0.02). Thus, DBT screening had potential for reducing interval cancer rate in the OTST from 2.1/1000 (51/24,301) to 1.6/1000 (38/24,301).

## Discussion

There is lack of knowledge from prospective trials regarding false-negative interpretations at double reading DBT versus double reading DM. The OTST found that DBT double reading detected significantly more cancers than DM but overall discordant rate (cancer missed by one reader) for screening-detected cancers was comparable (DBT 31% [71/227] vs. DM 30% [52/175], *p* = 0.81). Discordant rate for spiculated cancers detected by both modes was significantly lower for DBT (DBT 19% [20/106] vs. DM 36% [38/106], *p* = 0.003) but was high (57% [28/49]) for DBT-only detected lesions. Rate of “discordant miss” (highly suspicious score by one reader and false-negative by the other) was more than twice as high for DBT. Retrospective classification of DBT screening exams classified 95% (39/41) of discordant cancers detected by both modes, 100% (30/30) of discordant DBT-only detected cancers, and 25% (13/51) of interval cancers as “actionable,” with a potential reduction of DBT interval cancer rate by 25%. Reading time for “actionable” discordant DBT-only detected spiculated cancers revealed large differences between true-positives and false-negatives, nearly half false-negatives having reading times shorter than true-negatives.

Our results neither confirm decreased DBT interobserver variability previously reported [5] nor reduced usefulness of double reading with DBT [8]. The DBT discordant rate was high (31% [48/155]) for spiculated cancers. DBT may demonstrate small underlying masses in cancers presenting as architectural distortion on DM [15], and higher conspicuity and visibility of desmoplastic lesions on DBT have improved detection and reader confidence [16, 17]. Nevertheless, small spiculated cancers are occasionally seen on only one DBT view and a few slices [15, 18] causing such lesions to represent a perception and interpretation challenge [19]. The high discordant rate in our study for DBT-only detected spiculated cancers (57% [28/49]) agrees with this experience. There have been no previous studies presenting discordant interpretations in DBT screening. Our 2D discordant rate is comparable with results reported in 2D population-based screening using similar rating scale [20]. Regarding high discordant rate for cancers with calcifications using DBT, we suggest this is caused by interpretation rather than perception error.

Missed screening cancer might be caused by perception (detection) or cognitive (interpretation) error. Perceptual errors, considerably more common than interpretation errors, occur when an abnormality is determined to be present in retrospect but was not detected prospectively. Reasons for these missed cancers include poor lesion conspicuity, radiologist fatigue, and workplace distraction or interruptions [4]. The most commonly missed and misinterpreted lesions include benign-appearing masses, one-view findings, developing asymmetries, subtle calcifications, and architectural distortion [21]. Architectural distortion is one of the most frequently missed signs of breast cancer, and DBT may demonstrate suspicious lesions that are occult to DM [22]. A missed cancer at DBT seems to be related to interpretative error regarding clearly visible lesions, a problem that may be reduced with increased experience [23]. Several of the commonly missed lesions listed above might be correctly diagnosed using supplemental imaging (including fine-focus magnification views or ultrasound) or even at short-term follow-up. It is, however, important to keep in mind that the decision at our consensus meetings is purely binary (case recalled or dismissed) without including any report or description, and short-term follow-up for indeterminate findings (corresponding to BI-RADS category 3) is never used.

Interval cancers and long-term outcomes after DBT screening have been a hot topic. Our retrospective analysis classifying 13 of 51 prior DBT screening exams as “actionable” with a potential to reduce interval cancer rate by 25% is of interest. To the best of our knowledge, only the Malmø trial [3] has reported a reduction of interval cancer rate in DBT screening. A recently published large retrospective US study found no significant difference in the rates of screening-detected advanced cancers or interval cancers [24]. The prospective Italian RET trial reported that DBT screening in younger women (age 45–49) and women with dense breast having higher cancer detection at baseline was followed by a lower incidence of interval cancers [25].

Our median reading times for true-negative DBT exams are comparable with reported 56–77 s in other screening studies [26–28], although shorter times have been reported [29]. Longer reading time for true-positives is expected, but longer times for false-negatives are more interesting because readers might have seen the suspicious lesion but after some consideration dismissed the finding. A median (or mean) value does not reflect the complexity of missed cancers, and we observed large variation in reading times among radiologists. Discrepancy for pairs of discordant DBT-only detected spiculated cancers may indicate that some false-negatives with long reading times represent interpretation errors whereas short reading times represent perception errors due to fast reading. Rush and fatigue might cause readers to rely too much on poor 2D cancer conspicuity with incomplete analysis of DBT. We noticed that poor cancer visibility on DM was associated with discordant rates at DBT, but our numbers are insufficient for final conclusions.

Screening studies comparing DBT versus DM have so far paid little attention to the image interpretation process itself. Suboptimal reading environment, heavy workload using batch reading, and incomplete use of the complex hanging protocols with many DBT images may all cause cancers to be missed. Increased number of false-negatives throughout batch and reduced reading time for later image positions within batch was reported in DM screening [30]. High-volume DBT screening using batch reading requires greater cognitive resources than DM and might exacerbate association between fatigue and reader performance [31]. An experimental study found that readers were beginning to show signs of visual fatigue after 20 DBT cases [32]. Small cancers are often difficult to identify, and one study reported a higher proportion of cancers detected on only one view at DBT (10.5%) than at DM screening (4.7%) [33]. Small spiculated cancers seen on only a few slices require a systematic use of hanging protocols of both views in order not to miss cancers even in women with fatty breasts (Fig. 3). Simplified hanging protocols using slabs only may reduce reading time but have a negative impact on sensitivity [34]. On the other hand, DBT screening strategies using artificial intelligence (AI-based) systems have the potential to improve cancer detection and reduce reading time and workload, and could allow for more cost-effective breast cancer screening with DBT [35].

Our study has several limitations. First, it was conducted at a single institution with equipment from a single vendor. Second, radiologists often carried out image evaluation during overtime, and fatigue and rush may have contributed to false-negatives. Third, the study used no short-term follow-up which might have influenced decision-making. Fourth, the two DBT arms were not identical using DM in one and SM in the other arm in combination with DBT, but studies have shown comparable diagnostic accuracy using these two reading modes [11, 36].

In conclusion, overall discordant rate was comparable for DBT and DM, but significantly lower for DBT in spiculated cancers detected at both modes. Retrospective analysis of screening exams at baseline showed that DBT screening had a potential to reduce the interval cancer rate. DBT-only detected spiculated lesions revealed high discordant rate. False-negatives remain a major challenge in DBT screening. Most false-negative or discordant DBT exams were retrospectively classified as “actionable.” High-volume DBT screening using batch reading is a demanding task, and future studies should consider how implementation of artificial intelligence–based computer-aided detection and simplified hanging protocols could contribute to reduce workload and improved accuracy.

## Abbreviations

CAD: Computer-aided detection
DBT: Digital breast tomosynthesis
DM: Digital mammography
OTST: Oslo Tomosynthesis Screening Trial
SM: Synthetic mammography

## Declarations

### Guarantor

The scientific guarantor of this publication is Per Skaane.

### Statistics and biometry

One of the authors (Bjørn Helge Østerås) has significant statistical expertise.

### Study subjects or cohorts overlap

Some study subjects or cohorts have been previously reported in:

Skaane P, et al Radiology 2013;267(1):47–56 [ref. 9]

Skaane P, et al Eur Radiol 2013;23(8):2061–2071 [ref. 10]

Skaane P, et al Radiology 2014;271(3):655–663 [ref. 11]

Skaane P, et al Breast Cancer Res Treat 2018;169(3):489–496 [ref. 12]

Skaane P, et al Radiology 2019;291:23–30 [ref. 13]

Østerås BH, et al Radiology 2019;293:60–68 [ref. 14]

We have listed these references in “Materials and methods” (“OTST results have been previously reported [9–14]”). This article presents unpublished data from the OTST, and in addition, results from a retrospective reader study of the false-negative and interval cancer cases not earlier published.

### Methodology

• prospective

• diagnostic or prognostic study

• performed at one institution

## References

[1] Marinovich ML, Hunter KE, Macaskill P (2018). Breast cancer screening using tomosynthesis or mammography: a meta-analysis of cancer detection and recall. J Natl Cancer Inst.

[2] Houssami N, Zackrisson S, Blazek K (2021). Meta-analysis of prospective studies evaluating breast cancer detection and interval cancer rates for digital breast tomosynthesis versus mammography population screening. Eur J Cancer.

[3] Johnson K, Lång K, Ikeda DM (2021). Interval breast cancer rates and tumor characteristics in the prospective population-based Malmø Breast Tomosynthesis Screening Trial. Radiology.

[4] Korhonen KE, Weinstein SP, McDonald ES (2016). Strategies to increase cancer detection: review of true-positive and false-negative results at digital breast tomosynthesis screening. Radiographics.

[5] Dibble EH, Lourenco AP, Baird GL (2018). Comparison of digital mammography and digital breast tomosynthesis in the detection of architectural distortion. Eur Radiol.

[6] Hoff SR, Abrahamsen AL, Samset JH (2012). Breast cancer: missed interval and screening-detected cancer at full-field digital mammography and screen-film mammography – results from a retrospective review. Radiology.

[7] Hofvind S, Geller BM, Rosenberg RD (2009). Screening-detected breast cancers: discordant independent double reading in a population-based screening program. Radiology.

[8] Caumo F, Zorzi M, Brunelli S (2018). Digital breast tomosynthesis with synthesized two-dimensional images versus full-field digital mammography for population screening: outcomes from the Verona screening program. Radiology.

[9] Skaane P, Bandos AI, Gullien R (2013). Comparison of digital mammography alone and digital mammography plus tomosynthesis in a population-based screening program. Radiology.

[10] Skaane P, Bandos AI, Gullien R (2013). Prospective trial comparing full-field digital mammography (FFDM) versus combined FFDM and tomosynthesis in a population-based screening programme using independent double reading with arbitration. Eur Radiol.

[11] Skaane P, Bandos AI, Eben EB (2014). Two-view digital breast tomosynthesis screening with synthetically reconstructed projection images: comparison with digital breast tomosynthesis with full-field digital mammographic images. Radiology.

[12] Skaane P, Sebuødegård S, Bandos AI (2018). Performance of breast cancer screening using digital breast tomosynthesis: results from the prospective population-based Oslo Tomosynthesis Screening Trial. Breast Cancer Res Treat.

[13] Skaane P, Bandos AI, Niklason LT (2019). Digital mammography versus digital mammography plus tomosynthesis in breast cancer screening: the Oslo Tomosynthesis Screening Trial. Radiology.

[14] Østerås BH, Martinsen ACT, Gullien R (2019). Digital mammography versus breast tomosynthesis: impact of breast density on diagnostic performance in population-based screening. Radiology.

[15] Durand MA, Wang S, Hooley RJ (2016). Tomosynthesis-detected architectural distortion: management algorithm with radiologic-pathologic correlation. Radiographics.

[16] Alshafeiy TL, Nguyen JV, Rochman CM (2018). Outcome of architectural distortion detected only at breast tomosynthesis versus 2D mammography. Radiology.

[17] Bahl M, Lamb LR, Lehman CD (2017). Pathologic outcomes of architectural distortion on digital 2D versus tomosynthesis mammography. AJR Am J Roentgenol.

[18] Korhonen KE, Conant EF, Cohen EA (2019). Breast cancer conspicuity on simultaneously acquired digital mammographic images versus digital breast tomosynthesis images. Radiology.

[19] Bernardi D, Houssami N (2017). Breast cancers detected in only one of two arms of a tomosynthesis (3D-mammography) population screening trial (STORM-2). Breast.

[20] Martiniussen MA, Sagstad S, Larsen M (2022). Screen-detected and interval breast cancer after concordant and discordant interpretations in a population based screening program using independent double reading. Eur Radiol.

[21] Lamb LR, Fonseca MM, Verma R (2020). Missed breast cancer: effects of subconscious bias and lesion characteristics. Radiographics.

[22] Ray KM, Turner E, Sickles EA (2015). Suspicious findings at digital breast tomosynthesis occult to conventional digital mammography: imaging features and pathology findings. Breast J.

[23] Lång K, Andersson I, Zackrisson S (2014). Breast cancer detection in digital breast tomosynthesis and digital mammography – a side-by-side review of discrepant cases. Br J Radiol.

[24] Sprague BL, Coley RY, Lowry KP (2023). Digital breast tomosynthesis versus digital mammography screening performance on successive screening rounds from the Breast Cancer Surveillance Consortium. Radiology.

[25] Pattacini P, Nitrosi A, Rossi PG (2022). A randomized trial comparing breast cancer incidence and interval cancers after tomosynthesis plus mammography versus mammography alone. Radiology.

[26] Houssami N, Lockie D, Clemson M (2019). Pilot trial of digital breast tomosynthesis (3D mammography) for population-based screening in BreastScreen Victoria. Med J Aust.

[27] Bernardi D, Ciatto S, Pellegrini M (2012). Application of breast tomosynthesis in screening: incremental effect on mammography acquisition and reading time. Br J Radiol.

[28] Pattacini P, Nitrosi A, Rossi PG (2018). Digital mammography versus digital mammography plus tomosynthesis for breast cancer screening: the Reggio Emilia Tomosynthesis randomized trial. Radiology.

[29] Hofvind S, Holen ÅS, Aase HS (2019). Two-view digital breast tomosynthesis versus digital mammography in a population-based breast cancer screening programme (To-Be): a randomised, controlled trial. Lancet Oncol.

[30] Backmann HA, Larsen M, Danielsen AS (2021). Does it matter for the radiologists’ performance whether they read short or long batches in organized mammographic screening?. Eur Radiol.

[31] Bernstein MH, Baird GL, Lourenco AP (2022). Digital breast tomosynthesis and digital mammography recall and false-positive rates by time of day and reader experience. Radiology.

[32] Roy D, Sharma N, Koh A (2020). Fatigue while reading digital breast tomosynthesis (DBT) cases: determination of fatigue onset based on blinks. Clin Radiol.

[33] Caumo F, Romanucci G, Hunter K (2018). Comparison of breast cancers detected in the Verona screening program following transition to digital breast tomosynthesis screening with cancers detected at digital mammography screening. Breast Cancer Res Treat.

[34] Iotti V, Rossi PG, Nitrosi A (2019). Comparing two visualization protocols for tomosynthesis in screening: specificity and sensitivity of slabs versus planes plus slabs. Eur Radiol.

[35] van Winkel SL, Rodriguez-Ruiz A, Appelman L (2021). Impact of artificial intelligence support on accuracy and reading time in breast tomosynthesis image interpretation: a multi-reader multi-case study. Eur Radiol.

[36] Abdullah P, Alabousi M, Ramadan S (2020). Synthetic 2D mammography versus standard 2D digital mammography: a diagnostic test accuracy systematic review and meta-analysis. AJR.

